# A four-lncRNA signature for predicting prognosis of recurrence patients with gastric cancer

**DOI:** 10.1515/med-2021-0241

**Published:** 2021-04-03

**Authors:** Qiang Chen, Zunqi Hu, Xin Zhang, Ziran Wei, Hongbing Fu, DeJun Yang, Qingping Cai

**Affiliations:** Department of Gastrointestinal Surgery, Changzheng Hospital, Naval Medical University, No. 415 Fengyang Road, Huangpu District, Shanghai 200003, China

**Keywords:** SVM–RFE, RF–OOB, PCA, lncRNA, ceRNA network, prognosis

## Abstract

**Purpose:**

This study aimed to develop a multi-long noncoding RNA (lncRNA) signature for the prediction of gastric cancer (GC) based on differential gene expression between recurrence and nonrecurrence patients.

**Methods:**

By repurposing microarray expression profiles of RNAs from The Cancer Genome Atlas (TCGA), we performed differential expression analysis between recurrence and nonrecurrence patients. A prognostic risk prediction model was constructed based on data from TCGA database, and its reliability was validated using data from Gene Expression Omnibus database. Furthermore, the lncRNA-associated competing endogenous RNA (ceRNA) network was constructed, namely, DIANA-LncBasev2 and starBase database.

**Results:**

We identified 363 differentially expressed RNAs (317 mRNAs, 18 lncRNAs, and 28 microRNAs [miRNAs]). Principal component analysis showed that the seven-feature lncRNAs screened by support vector machine–recursive feature elimination algorithm was more informative for predicting recurrence of GC in comparison with the eight-feature lncRNAs screened by random forest–out-of-bag algorithm. Four of the seven-feature lncRNAs including LINC00843, SNHG3, C21orf62-AS1, and MIR99AHG were chosen to develop a four-lncRNA risk score model. This risk score model was able to distinguish patients with high and low risk of recurrence, and was tested in two independent validation sets. The ceRNA network of this four-lncRNA signature included 10 miRNAs and 178 mRNAs. The mRNAs significantly related to the Wnt-signaling pathway and relevant biological processes.

**Conclusion:**

A useful four-lncRNA signature recurrence was established to distinguish GC patients with high and low risk of recurrence. Regulating the relevant miRNAs and Wnt pathway might partly affect GC metastasisby.

## 1 Introduction

Gastric cancer (GC) is the fifth common cancer characterized by high incidence and mortality [1]. Current management strategies for GC mainly include surgical resection guided by endoscopic detection and chemotherapy or chemoradiotherapy as adjuvant therapy [2]. Despite advancements in treatments, GC patients have unsatisfactory prognosis [3]. Conventional tumor node metastasis (TNM) stage system is inadequate for outcome prediction of GC patients [4], and novel prognostic biomarkers are complementary and necessary for identifying potential high-risk GC patients and contribute to better outcome in GC patients.

Long noncoding RNAs (lncRNAs) play important regulatory roles in cancer biogenesis. A large number of lncRNAs have been shown to be dysregulated in GC, participate in gastric tumorigenesis and progression through interacting with DNA, RNA, and proteins [5,6]. Moreover, lncRNAs are associated with the prognosis of GC patients, and several lncRNAs-based signatures have been reported for outcome prediction [7,8,9]. High incidence of recurrence following curative resection is a primary cause of undesirable prognosis in patients with advanced GC [10,11]. Therefore, we utilized gene profiling data of recurrence and nonrecurrence GC patients from The Cancer Genome Atlas (TCGA) to explore aberrantly expressed lncRNAs associated with recurrence and develop an lncRNA-based signature for prognosis stratification of GC patients. Two validation data sets from Gene Expression Omnibus (GEO) were used to confirm the prognostic ability of the signature. Furthermore, we unraveled recurrence-related differentially expressed microRNAs (miRNAs) and mRNAs as well as to study the relationships between signature lncRNAs, miRNAs, and potential targeted miRNAs by constructing competing endogenous RNA (ceRNA) network, thereby providing insights into the regulatory mechanisms of these signature lncRNAs in GC.

## 2 Methods

### 2.1 Data and preprocessing

We obtained RNA sequencing data (including mRNA and lncRNA) of 407 GC samples and miRNA sequencing data of 477 samples with the corresponding clinical information from the publicly accessible TCGA database (https://gdc-portal.nci.nih.gov/) based on Illumina HiSeq 2000 RNA Sequencing platform. A total of 287 samples with paired RNA and miRNA data as well as the corresponding overall survival (OS) information were selected as the training set. The detailed clinical information of samples in TCGA data sets is shown in Table S1.

Meanwhile, we launched a search in GEO database (https://www.ncbi.nlm.nih.gov/geo/) using “gastric cancer,” “stomach cancer,” and “homo sapiens” as the key words. Potential data sets were selected when the following criteria were met: the number of total samples ≥250 and the number of GC samples with corresponding clinical data ≥200. As a result, two data sets including GSE26253 and GSE62254 were chosen. GSE26253 [12] (GPL8432 Illumina HumanRef-8 WG-DASL v3.0 platform) contained gene expression data of 432 GC tissue samples with clinical information available (validation set 1), while GSE62254 [2] (Affymetrix Human Genome U133 Plus 2.0 Array platform) included gene profiling data of 300 GC tissue samples, among which 282 samples had the corresponding clinical data (validation set 2). The detailed clinical information of samples in GSE26253 and GSE62254 data sets is shown in Tables S2 and S3.

In order to supplement the information of targeted molecular therapy, we collected information about the targeted drugs from databases TCGA, xena.ucsc, and cbioporta. Unfortunately, we only got the information on whether the patients received “targeted treatment” or not; however, there were no specific information of targeted drug molecules. Meanwhile, the detailed clinical characteristics of the patients were analyzed and shown in Table S4. In order to exclude the effect of therapeutic schedule on the screening of the present lncRNAs, correlation analysis was used to eliminate the doubt in this aspect, and the results showed that four important lncRNAs were not related to radiotherapy, chemotherapy, and targeted therapy. Their detailed information is shown in Table S5.

Raw data were standardized as previously described by Chaudhary et al. [13]. HUGO Gene Nomenclature Committee (HGNC) [14] repository (http://www.genenames.org/) enrolled 4,313 lncRNAs, 19,197 protein-coding genes, and 1,914 miRNAs. Using the HGNC database, we annotated 13,105 mRNAs, 1,051 lncRNAs, and 413 miRNAs from the abovementioned data sets, according to RefSeq ID information.

### 2.2 Differential expression analysis between recurrence and nonrecurrence GC samples

We screened differentially expressed RNAs (DERs) including lncRNAs, mRNAs, and miRNAs between recurrence and nonrecurrence samples in the training set, using FDR <0.05 and |log_2_ FC| > 0.263 as a selection threshold. The identified significant lncRNAs, miRNAs, and mRNAs consequently underwent two-way hierarchical clustering analysis based on centered Pearson correlation algorithm [15].

### 2.3 Prognostic model building and validation

In order to identify recurrence-related feature lncRNAs from the preselected differentially expressed lncRNAs, we employed and compared the support vector machine–recursive feature elimination (SVM–RFE) and random forest–out-of-bag (RF–OOB) algorithms for performance. With regard to SVM–RFE [16] algorithm (100-fold cross validation), the lncRNAs subset with the best accuracy was chosen to be the signature lncRNAs. Using RF–OOB [17] algorithm, the subset of lncRNAs with the minimal value of OOB error was selected to be the optimal feature lncRNAs. Principal component analysis (PCA) method was applied to compare the performances of the two algorithms. The feature lncRNAs identified by the superior approach was applied in further analysis.

Using training set, we did univariable Cox regression analysis on the identified recurrence-related signature lncRNAs. The significant lncRNAs were further included in multivariable Cox regression analysis. log-rank *p* value <0.05 defined the significance. The lncRNAs that were independent prognostic indicators were selected to construct a prognostic score formula as following:

\text{Risk}\hspace{.5em}\text{score}\hspace{.5em}\text{(RS)}=\sum {\beta }_{\text{lncRNAn}}\times {\text{Exp}}_{\text{lncRNAn}}]

where here *β*_lncRNA_ suggests multivariable Cox regression coefficient of lncRNAn; Exp_lncRNAn_ indicates the expression value of lncRNAn.

We calculated the risk score for each patient in the training set based on the formula. Patients in the training set were categorized by the median risk score into a high-risk group and a low-risk group. Similarly, the lncRNAs-based risk score formula was applied to distinguish patients in two validation sets. Recurrence-free survival (RFS) time of two risk groups was compared using Kaplan–Meier method and log-rank test. Sensitivity and specificity of the risk score model were assessed using the ROC curve.

Using the training set data, we conducted uni- and multivariable Cox regression analysis to evaluate the association of clinical features and risk score model status with RFS time of patients. Integrating independent prognostic clinical features with risk score status, nomogram was built; and the calibration curves were plotted to ascertain its predictive performance.

### 2.4 Statistical analysis

A wide range of packages in R software (version 3.4.1) were utilized for bioinformatics and statistical analyses of our study: limma package (version 3.34.7) for differential expression analysis; pheatmap package (version 1.0.8) for two-way hierarchical clustering analysis; e1071 [18] (version1.7-1, https://cran.r-project.org/web/packages/e1071); caret [19] (version 6.0-76, https://cran.r-project.org/web/packages/caret) packages for SVM–RFE method; bootstrap algorithm of randomForest [20] package (https://cran.r-project.org/web/packages/randomForest/) for RF–OOB method; psych [21] package (version 1.8.12) for PCA; survival package (http://bioconductor.org/packages/survivalr/) for uni- and multivariable Cox regression analysis; Kaplan–Meier curves; pROC package (https://cran.r-project.org/web/packages/pROC/index.html) for ROC curve analysis; and rms package (version 5.1-2) for nomogram building.

### 2.5 Construction of lncRNA-associated ceRNA network

For dissecting the underlying molecular mechanisms of the prognostic signature lncRNAs in GC biology, we designed a three-phase study. Initially, the relations between the signature lncRNAs with the differentially expressed miRNAs were predicted based on DIANA-LncBasev2 [22] database. The negatively correlated lncRNA–miRNA pairs were selected to build an lncRNA–miRNA network.

Second, starBase database (version 2.0, http://starbase.sysu.edu.cn/) was used for the prediction of potential target mRNAs of the miRNAs included in the lncRNA–miRNA network. starBase program compiles prediction results from TargetScan, PicTar, RNA22, PITA, and miRanda [23]. The differentially expressed mRNAs were mapped to the predicted target mRNAs that were enrolled in at least three of the five programs. The miRNA–mRNA pairs with negative correlation were selected to develop an miRNA–mRNA network.

Third, an lncRNA–miRNA–mRNA network was built with predicted lncRNA–miRNA and miRNA–mRNA pairs with negative correlation. Networks were visualized with Cytoscape [24] software (version 3.6.1). All mRNAs in the networks were subject to gene ontology (GO) function and kyoto encyclopedia of genes and genomes (KEGG) pathway enrichment analysis by Enrichr [25] tool (http://amp.pharm.mssm.edu/Enrichr/). *P* value <0.05 was considered significant.

## 3 Results

### 3.1 Identification of differentially expressed mRNAs, lncRNAs, and miRNAs between recurrence and nonrecurrence GC patients

**Figure 1:**
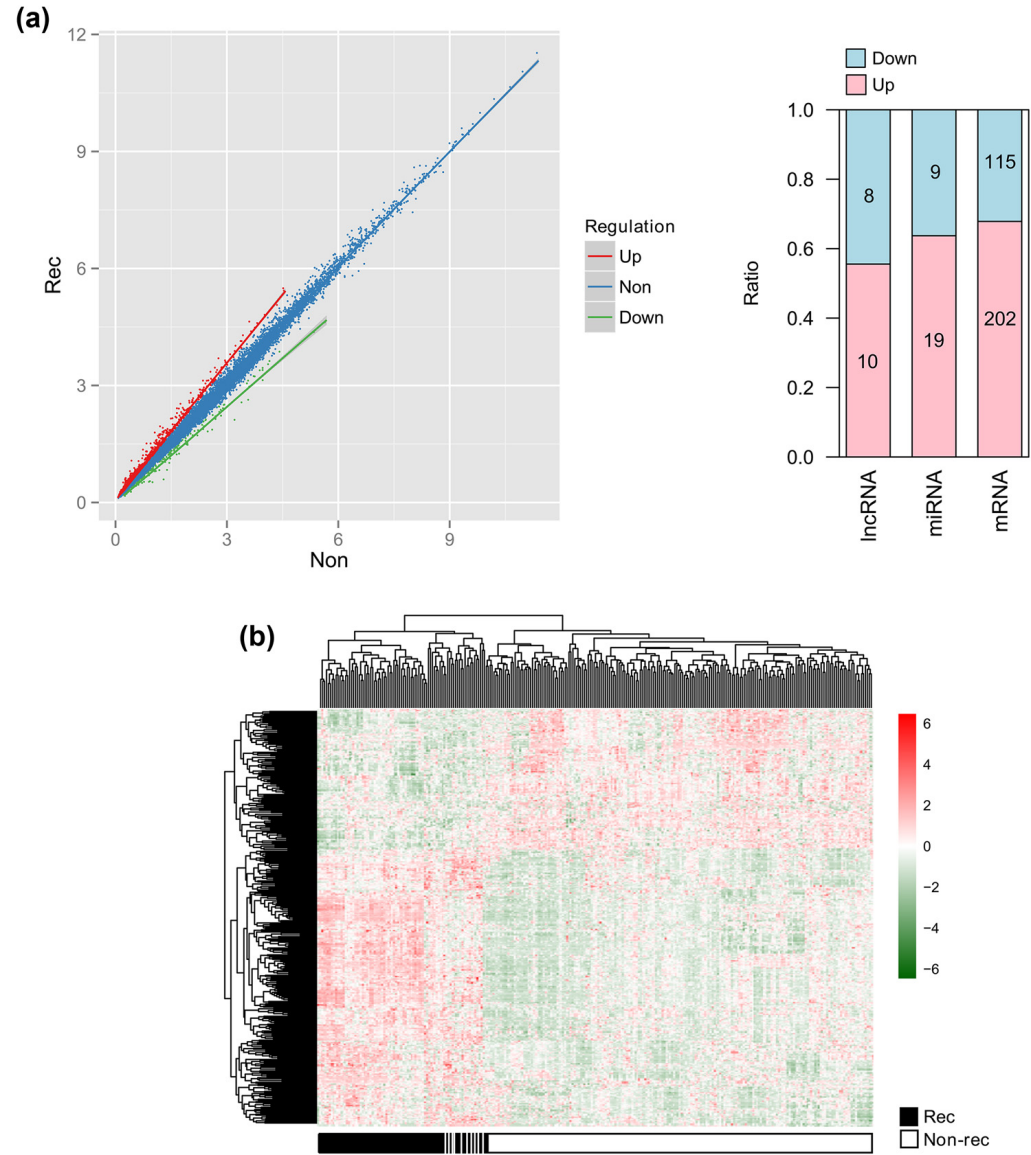
Differentially expressed lncRNAs, miRNAs, and mRNAs of recurrence and nonrecurrence patients. (a) Left image is a scatterplot for differentially expressed RNAs. Lateral axis represents nonrecurrence samples; vertical axis represents recurrence samples. Upregulated, downregulated, and nonregulated RNAs are shown in red, green, and blue spots, respectively. Right image shows ratios of up- and downregulated RNAs. (b) Heatmap displays differentially expressed RNAs in recurrence and nonrecurrence samples.

The training set (*N* = 287) consisted of 61 recurrence patients and 226 nonrecurrence patients. Totally 363 DERs between the recurrent and nonrecurrent patients were identified by performing differential gene expression analysis, which were consisted of 317 mRNAs (115 downregulated and 202 upregulated mRNAs; Table S6), 18 lncRNAs (8 downregulated and 10 upregulated lncRNAs; Table S7), and 28 miRNAs (9 downregulated and 19 upregulated miRNAs; Table S8; Figure 1a and b).

### 3.2 Construction of a four-lncRNA prognostic score risk model

**Figure 2:**
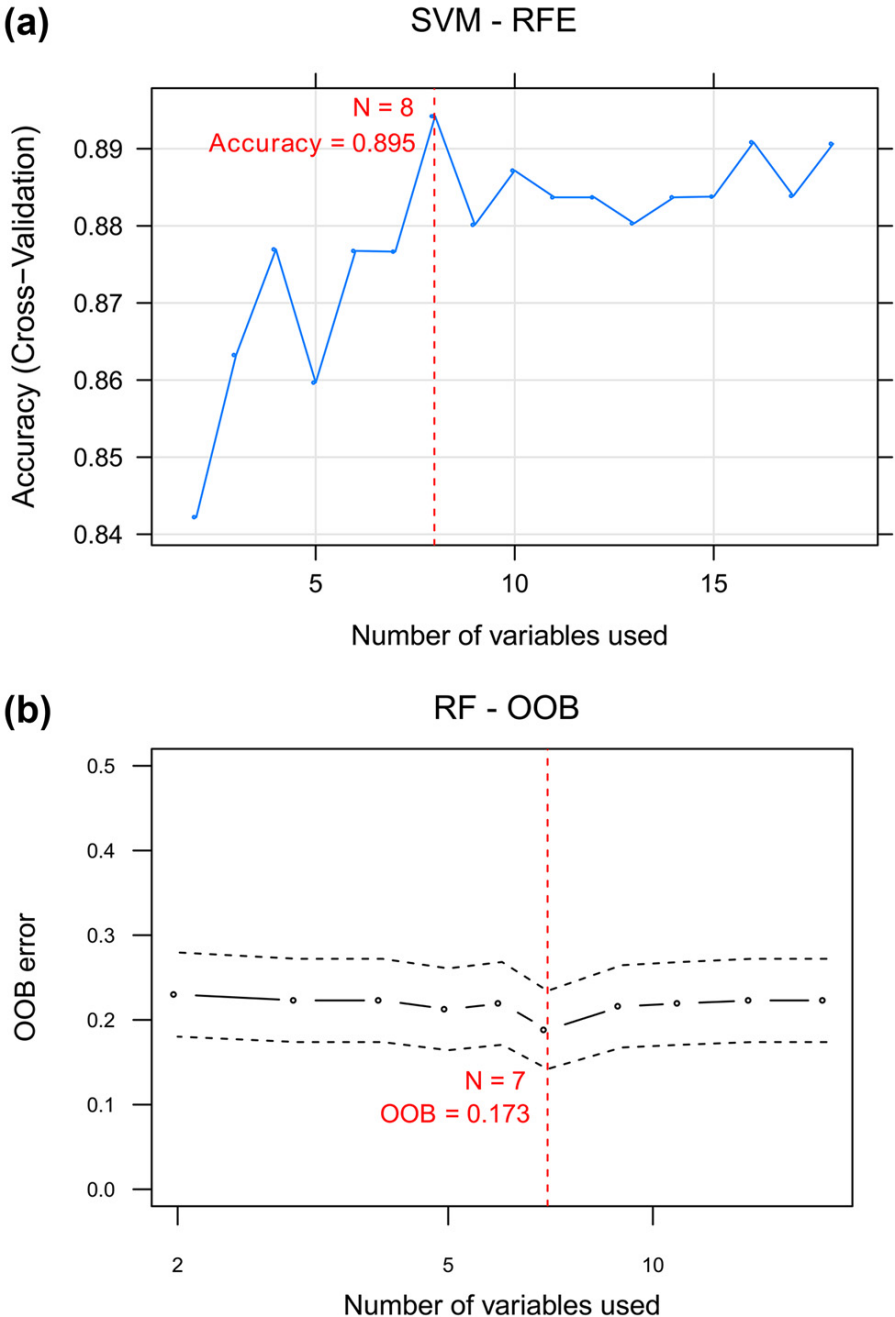
Feature lncRNAs selected by SVM–RFE (a) and RF–OOB (b). The red dashed line shows the number of lncRNAs achieving the highest accurracy (a) or the smallest OOB error (b).

**Figure 3:**
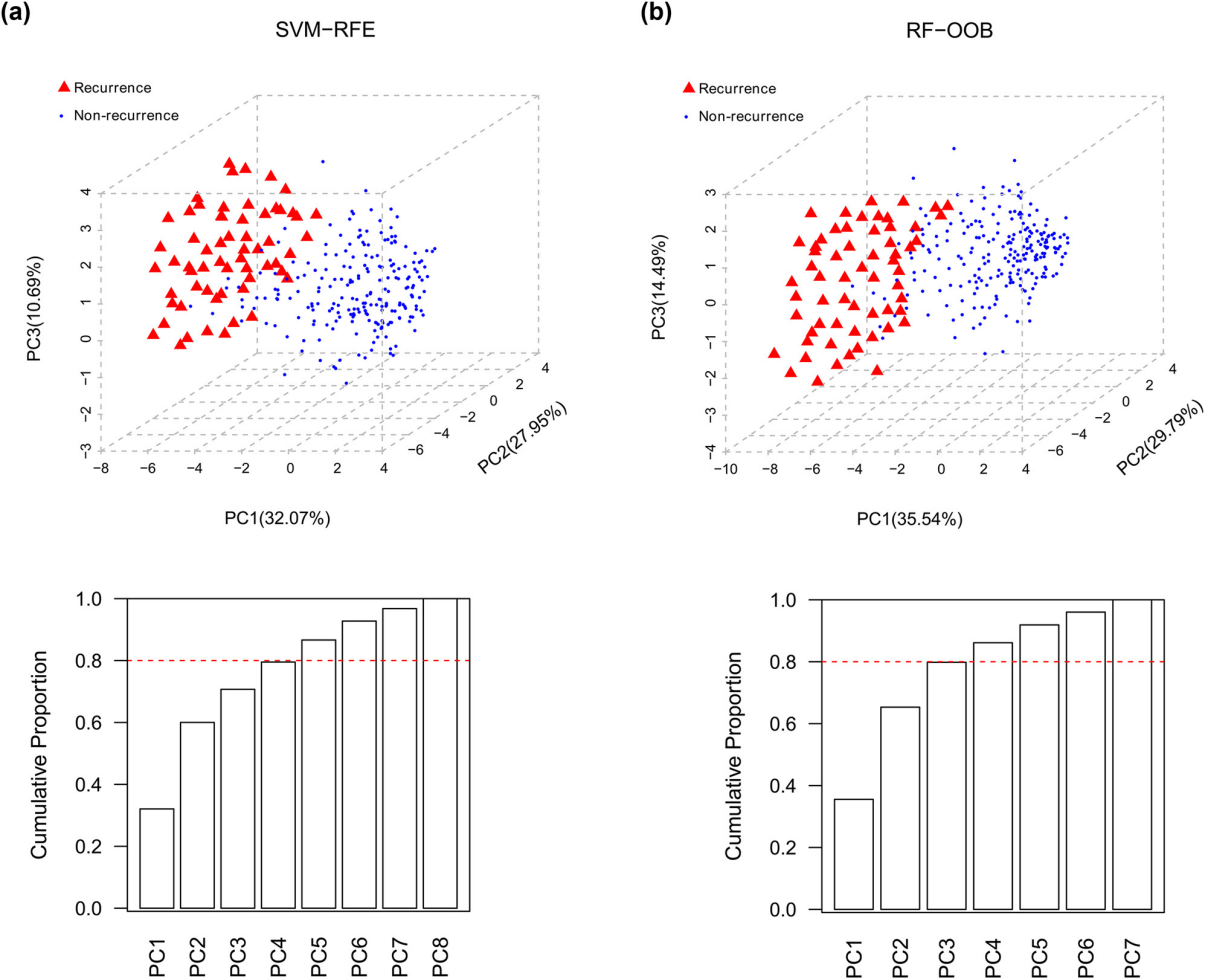
Comparative analysis of SVM–RFE (a) and RF–OOB (b) by PCA approach. Upper images illustrate 3D PCA plot of PC1, PC2, and PC3. Recurrence and nonrecurrence samples are labeled in red triangles and blue balls, respectively. Below images exhibit cumulative contribution of different PCs. The red dashed line indicates cumulative proportion of 80%.

We used SVM–RFE and RF–OOB algorithms to select feature lncRNAs informative of recurrence based on the abovementioned differentially expressed lncRNAs. Using SVF-RFM algorithm, we obtained a combination of eight-feature lncRNAs when achieving the highest accuracy = 0.895 (Figure 2a, Table S9). A set of seven lncRNAs was determined by RF–OOB algorithm (minimal OOB error = 0.173; Figure 2b; Table S10). The two groups of feature lncRNAs were compared by PCA. Regarding the eight-feature lncRNAs identified by SVM–RFE, the top four principal components in PCA approximately account for 80% of the total observed variances (Figure 3a). In contrast, the top three principal components for the seven-feature lncRNAs selected by RF–OOB algorithm could describe almost 80% of the total observed variances (Figure 3b). These results suggested that the seven-feature lncRNAs were more indicative of recurrence than the eight-feature lncRNAs and were thus used in further analysis.

By subjecting the seven-feature lncRNAs to a univariable Cox regression analysis, we found six prognosis-related lncRNAs (*p* value <0.05), which further underwent multivariable Cox regression analysis. Four lncRNAs (LINC00843, SNHG3, C21orf62-AS1, and MIR99AHG), which were independent predictors of prognosis, were depicted in Table 1. A risk score formula was created based on the expression of the four signature lncRNAs for prognosis prediction as follows:

\begin{array}{c}\text{Risk}\hspace{.25em}\text{score}=(-1.0637)\times {\text{Exp}}_{\text{LINC}00843}+(-0.4163)\times {\text{Exp}}_{\text{SNHG}3}+(1.4731)\times {\text{Exp}}_{\text{C}21\text{orf}62\text{-AS}1}\\ \hspace{5.5em}+(0.2341)\times {\text{Exp}}_{\text{MIR}99\text{AHG}}\end{array}]

**Table 1:** Characteristics of four independent prognostic lncRNAs

ID	Coefficient	*P* value	HR	95% CI
LINC00843	−1.0637	6.71 × 10^−3^	0.345	0.111–0.878
SNHG3	−0.4163	8.97 × 10^−3^	0.659	0.208–0.967
C21orf62-AS1	1.4731	9.17 × 10^−3^	4.363	1.788–7.171
MIR99AHG	0.2341	4.96 × 10^−2^	1.264	1.045–2.477

**Figure 4:**
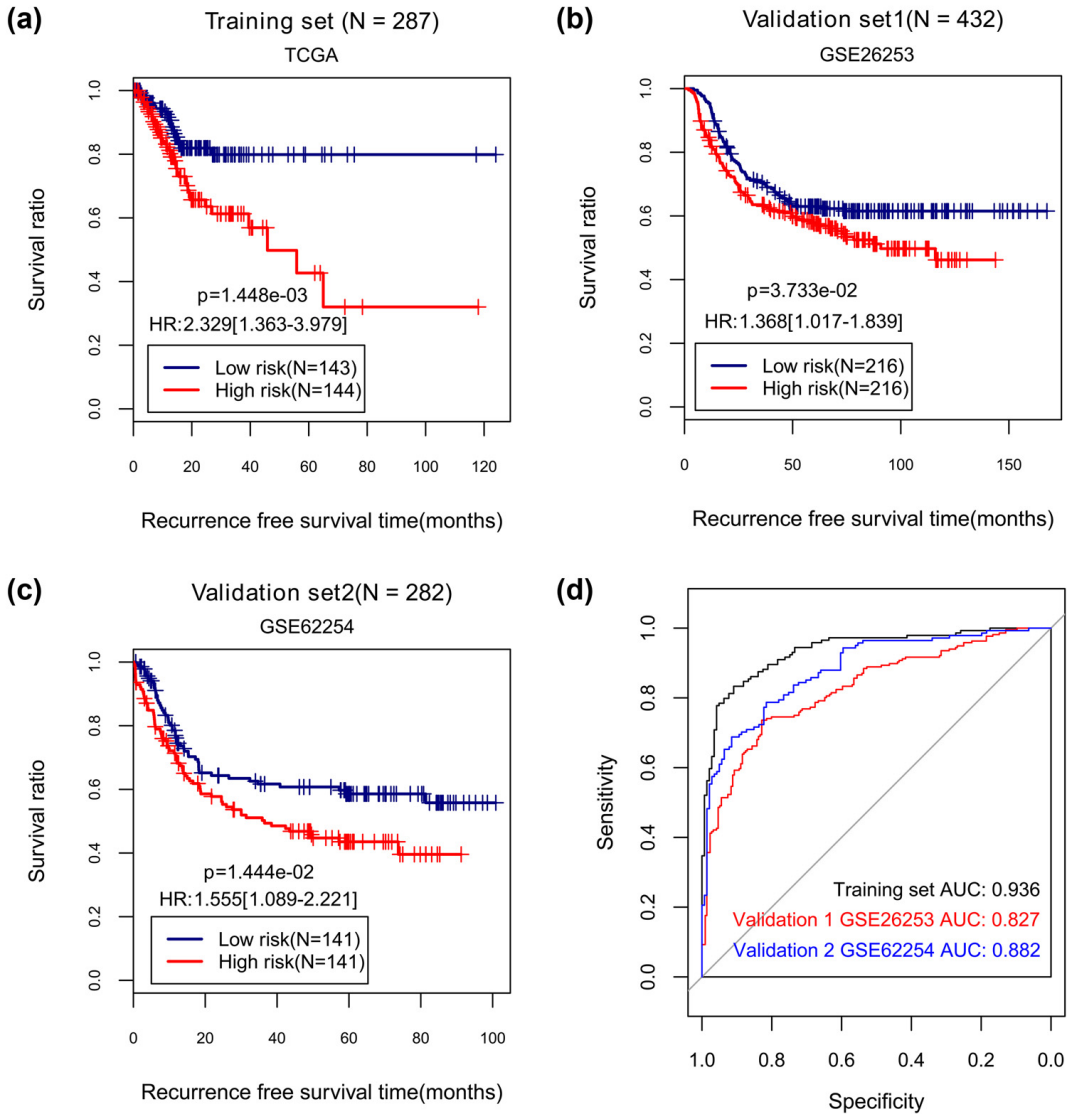
Kaplan–Meier estimates of recurrence-free survival and ROC curves for patients in training set and two validation sets by the four-lncRNA signature. (a–c) Kaplan–Meier curves for training set, validation set 1, and validation set 2. (d) ROC curves and AUC values of three data sets.

We calculated the risk score for each patient in the training set and ranked all the patients based on four-lncRNA signature. With the median risk score as the cutoff, the training set was categorized into a high-risk group (*N* = 144) and a low-risk group (*N* = 143). Significantly longer RFS time was observed in the low-risk patients relative to the high-risk patients (*p* value = 1.448 × 10^−3^, HR = 2.329[1.363–3.979]; Figure 4a). We validated the four-lncRNA signature risk score in two validation sets. As shown in Figure 4b and c, either validation set was divided by the risk score into two risk groups with statistical significance in RFS time (validation set 1: *p* value = 3.733 × 10^−2^, HR = 1.368[1.017–1.839]; validation set 2: *p* value = 1.444 × 10^−2^, HR = 1.555[1.089–2.221]). The risk score of each sample in the data set TCGA, GSE26253, and GSE62254 is shown in Table S11(1–3), respectively. The AUC values of ROC curves were 0.936, 0.827, and 0.822 for training set, validation set 1, and validation set 2, separately (Figure 4d). These observations demonstrated robust predictive performance of the four-lncRNA signature risk score in GC.

### 3.3 Building nomogram based on gender, histologic grade, and risk score model status

By performing univariable Cox regression analyses to assess the relationship of clinical variables and risk score model status with RFS time of patients in the training set, gender (HR = 1.761, 95% CI = 0.967–1.014, *p* value = 4.92 × 10^−2^), neoplasm histologic grade (HR = 2.181, 95% CI = 1.232–3.856, *p* value = 6.19 × 10^−3^), and the four-lncRNA risk score model status (HR = 2.329, 95% CI = 1.363–3.979, *p* value = 1.45 × 10^−3^) were statistically significant (Table 2). Furthermore, multivariable Cox regression analysis incorporating risk score model status with gender and neoplasm histologic grade was performed. Gender (HR = 1.735, 95% CI = 1.179–3.078, *p* value = 4.93 × 10^−2^), neoplasm histologic grade (HR = 2.063, 95% CI = 1.156–3.682, *p* value = 1.43 × 10^−2^), and risk score model status (HR = 2.059, 95% CI = 1.198–3.537, *p* value = 8.91 × 10^−3^) were found to be independent prognostic predictors (Table 2), indicating that prognostic value of the four-lncRNA risk score is independent of other clinical features.

**Table 2:** Uni- and multivariable Cox regression analysis of clinical features and risk score model status

Clinical characteristics	TCGA (*N* = 287)	Univariables Cox			Multivariables Cox		
		HR	95% CI	*p*	HR	95% CI	*p*
Age (years, mean ± sd)	65.06 ± 10.65	0.991	0.967–1.014	4.12 × 10^−1^	—	—	—
Gender (male/female)	181/106	1.761	0.994–3.117	**4.92 × 10** ^**−2**^	1.735	1.179–3.078	**4.93 × 10** ^**−2**^
Pathologic_M (M0/M1/–)	259/14/14	1.149	0.359–3.679	8.15 × 10^−1^	—	—	—
Pathologic_N (N0/N1/N2/N3/–)	89/77/58/53/10	1.151	0.918–1.440	2.24 × 10^−1^	—	—	—
Pathologic_T (T1/T2/T3/T4/–)	16/64/125/78/4	0.796	0.599–1.056	1.12 × 10^−1^	—	—	—
Pathologic_stage (I/II/III/IV/–)	41/95/121/19/11	1.029	0.763–1.387	8.53 × 10^−1^	—	—	—
Neoplasm histologic grade (G1/G2/G3/–)	5/101/172/9	2.181	1.232–3.856	**6.19 × 10** ^**−3**^	2.063	1.156–3.682	**1.43 × 10** ^**−2**^
Radiation therapy (Yes/No/–)	54/229/4	0.627	0.321–1.225	1.69 × 10^−1^	—	—	—
Targeted molecular therapy (Yes/No/–)	130/151/6	1.375	0.824–2.293	2.21 × 10^−1^	—	—	—
*Helicobacter pylori* infection (Yes/No/–)	15/107/165	0.314	0.119– 2.358	2.35 × 10^−1^	—	—	—
Residual tumor (R0/R1/R2/R3/–)	239/10/6/32	1.774	0.922–3.413	1.28 × 10^−1^	—	—	—
Chemotherapy (Yes/No/–)	136/151	1.313	0.772–2.231	3.13 × 10^−1^	—	—	—
RS model status (High/Low)	143/144	2.329	1.363–3.979	**1.45 × 10** ^**−3**^	2.059	1.198–3.537	**8.91 × 10** ^**−3**^
Recurrence (Yes/No)	61/226	—	—	—	—	—	—
Recurrence-free survival time (months, mean ± sd)	19.70 ± 18.48	—	—	—	—	—	—

Statistically significant data are indicated by bold for significant at *p* < 0.05.

**Figure 5:**
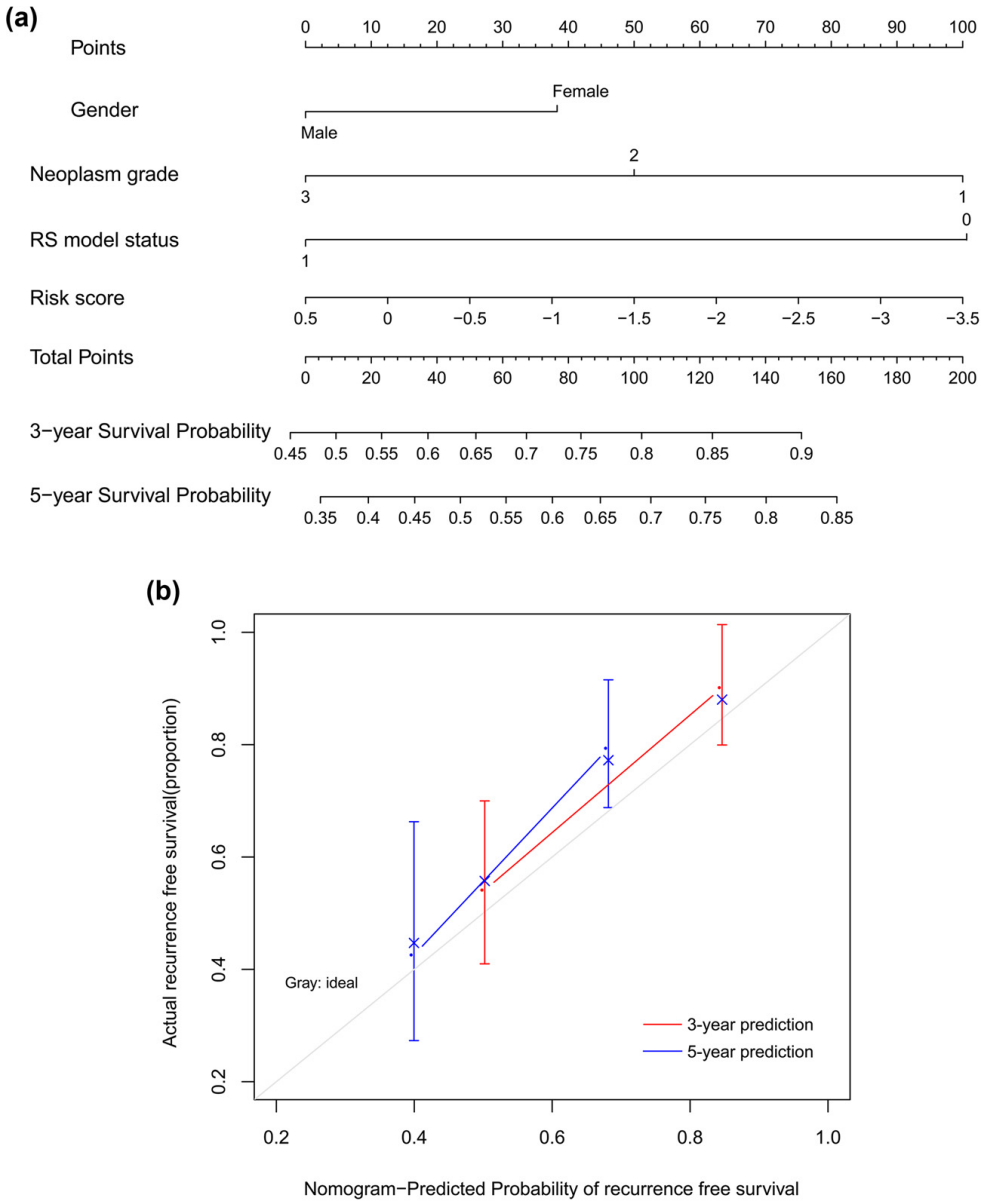
Nomogram to predict 3-year and 5-year probability of recurrence-free survival using TCGA set. (a) nomogram based on gender, neoplasm grade, and risk score model status. RS model status = four-lncRNA risk score model status. (b) calibration curves of actual and predicted probabilities.

A composite nomogram on the basis of gender, neoplasm histologic grade, and risk score status was established to predict the probability of 3-year and 5-year RFS (Figure 5a). Calibration plot for goodness of fit of the model exhibited good consistence between the predicted and actual RFS time (Figure 5b).

### 3.4 Characterization of ceRNA network of four signature lncRNAs and functional annotation

**Figure 6:**
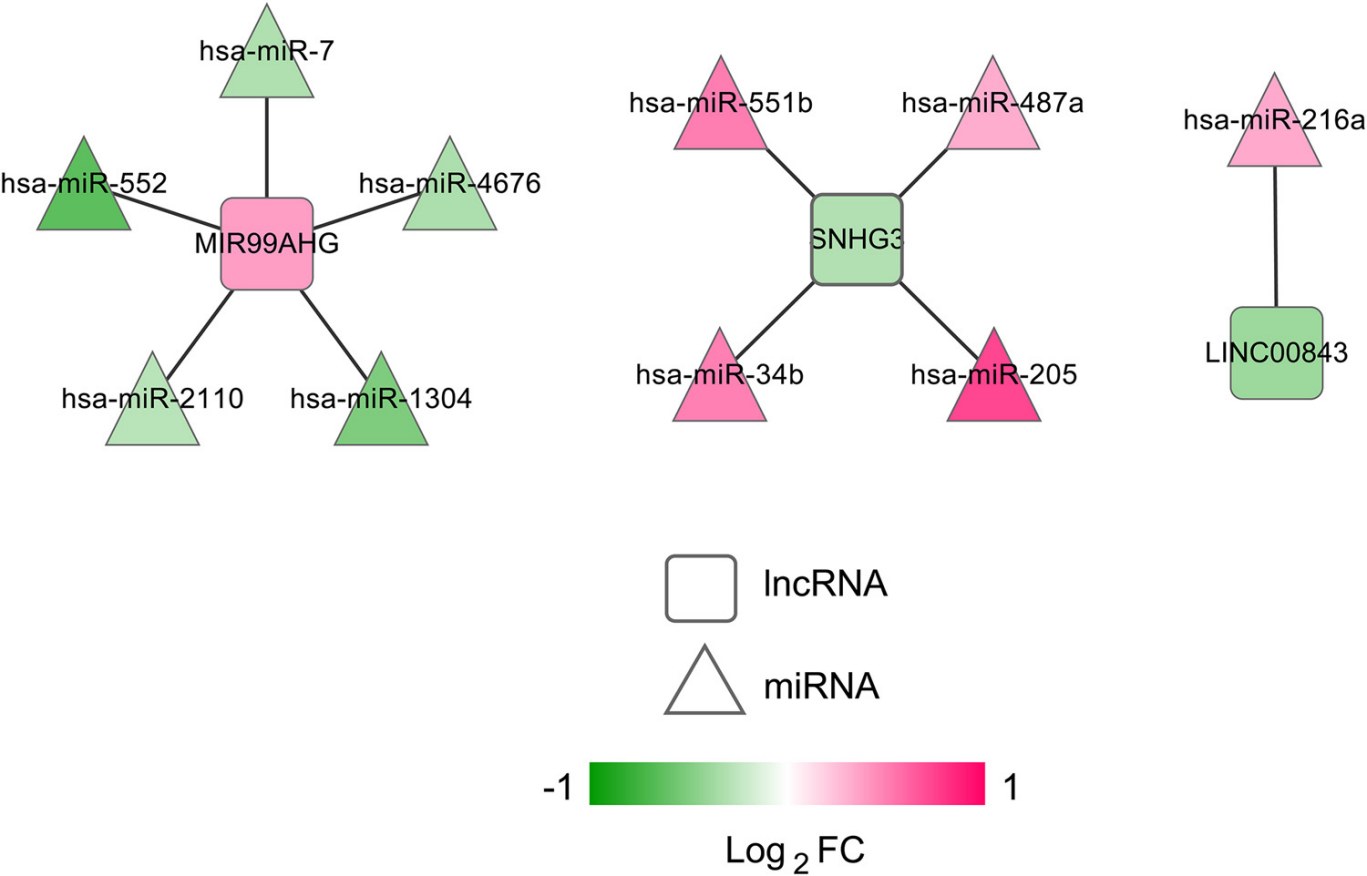
Signature lncRNA–miRNA network. Diamonds and triangles stand for lncRNAs and miRNAs, respectively.

**Figure 7:**
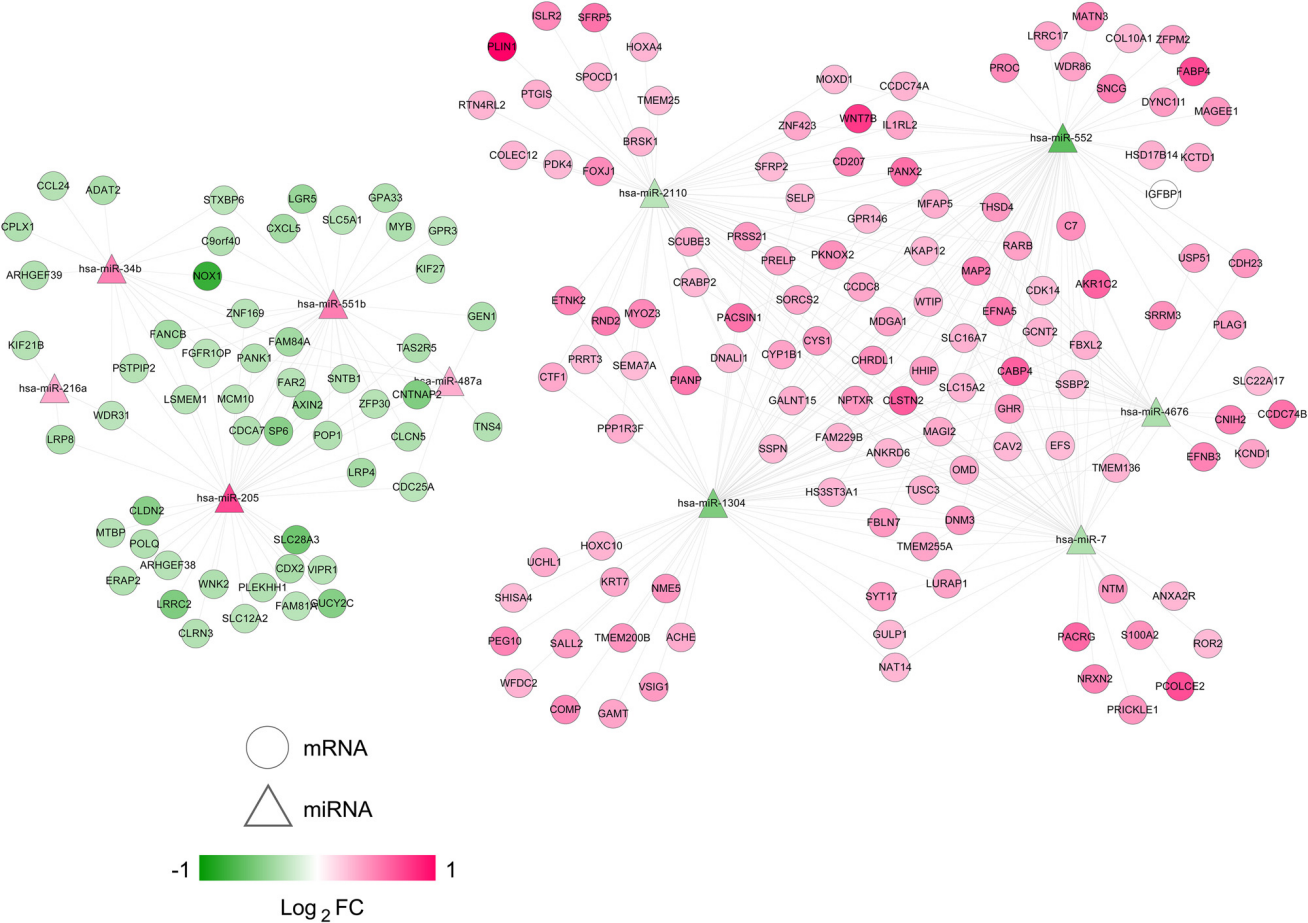
The miRNA–mRNA network. Balls and triangles denote mRNAs and miRNAs, respectively.

**Figure 8:**
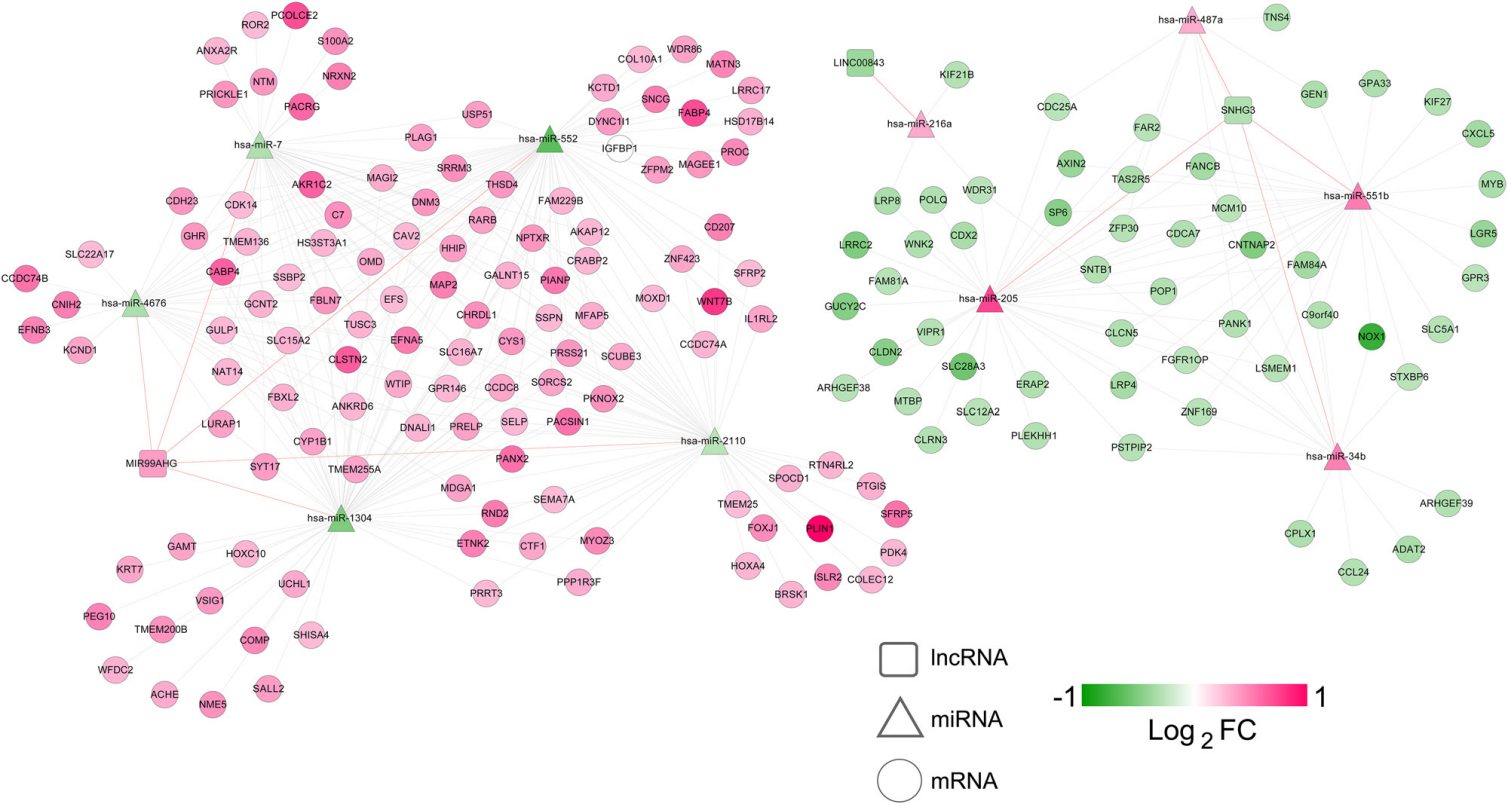
A ceRNA network of signature lncRNAs, miRNAs, and mRNAs. Diamonds, triangles, and balls represent signature lncRNAs, miRNAs, and mRNAs, separately. Red and black links suggest lncRNA–miRNA and miRNA–mRNA interactions, respectively.

Using DIANA-LncBasev2, we analyzed the correlations between the four signature lncRNAs and the differentially expressed miRNAs. As a result, we procured 10 negatively correlated lncRNA–miRNA pairs and constructed an lncRNA–miRNA network. There were 3 lncRNAs (upregulated MIR99AHG, downregulated LINC00843, and SNHG3) and 10 miRNAs (five upregulated and five downregulated miRNAs) in the network (Figure 6). As to the 10 miRNAs (miR-7, miR-552, miR-4676, miR-1304, miR-2110, miR-216a, miR-205, miR-487a, miR-551b, and miR-34b), we predicted potential target mRNAs by using starBase, which were further mapped by the 321 differentially expressed mRNAs. The resulting 351 miRNA–mRNA pairs with negative correlation were used to develop a miRNA–mRNA network. Figure 7 exhibited this network of 10 miRNAs and 178 mRNAs (54 downregulated and 124 upregulated mRNAs). Finally, the preselected negatively correlated lncRNA–miRNA pairs and miRNA–mRNA pairs were integrated into a ceRNA regulatory network. Three signature lncRNAs, 10 miRNAs, and 178 mRNAs were observed in the network (Figure 8). Their detailed information are shown in Tables S12 and S13.

The GO function and KEGG pathway-enrichment analysis for the mRNAs in the ceRNA network was conducted. These mRNAs were significantly enriched in 19 GO terms predominately related to Wnt-signaling pathway, cell proliferation, migration, and other biological processes (Table 3). Five significant KEGG-signaling pathways were noted, including Wnt-signaling pathway, cell adhesion molecules, GC, regulation of lipolysis in adipocytes, and steroid hormone biosynthesis pathways (Table 3). These results revealed that four signature lncRNAs-related ceRNA regulation participated in a variety of biological functions and signaling pathways in GC.

**Table 3:** Results of GO function and KEGG pathway enrichment analysis

Type	Term	Count of significantly enriched genes	*P* value
GO biology process	Regulation of canonical Wnt-signaling pathway	10	2.27 × 10^−5^
	Negative regulation of canonical Wnt-signaling pathway	7	3.82 × 10^−4^
	Regulation of cell migration	10	5.67 × 10^−4^
	Neuron projection morphogenesis	7	6.77 × 10^−4^
	Regulation of cell proliferation	16	9.86 × 10^−4^
	Negative regulation of Wnt-signaling pathway	7	9.91 × 10^−4^
	Cell morphogenesis involved in neuron differentiation	5	1.84 × 10^−3^
	Positive regulation of cell growth	5	1.92 × 10^−3^
	Wnt-signaling pathway	5	3.04 × 10^−3^
	Positive regulation of cell motility	6	5.55 × 10^−3^
	Positive regulation of cellular process	11	7.01 × 10^−3^
	Skeletal system development	5	1.02 × 10^−2^
	Positive regulation of cell proliferation	9	1.41 × 10^−2^
	Positive regulation of cell migration	6	1.47 × 10^−2^
	Extracellular matrix organization	6	1.72 × 10^−2^
	Positive regulation of multicellular organismal process	5	3.54 × 10^−2^
	Negative regulation of cell proliferation	7	4.47 × 10^−2^
	Proteolysis	6	4.69 × 10^−2^
KEGG pathway	Wnt-signaling pathway	7	5.43 × 10^−4^
	Cell adhesion molecules (CAMs)	4	4.08 × 10^−2^
	Gastric cancer	4	4.43 × 10^−2^
	Regulation of lipolysis in adipocytes	2	4.86 × 10^−2^
	Steroid hormone biosynthesis	2	4.98 × 10^−2^

## 4 Discussion

Although a growing number of prognostic lncRNAs for GC have been uncovered [26], some limitations are present, such as small number of lncRNAs, limited sample size, and insufficient validation. GC patients often experience recurrence following surgical resection [27]. To develop a recurrence-related multi-lncRNA signature for the prediction of RFS in GC patients, we repurposed the existing microarray data downloaded from TCGA to profile lncRNAs, miRNAs, and mRNAs in GC patients. A total of 363 DERs between recurrence and nonrecurrence patients were obtained, comprising 18 lncRNAs, 317 mRNAs, and 28 miRNAs.

Another highlight of this study was that we applied and compared SVM–RFE and RF–OOB to identify feature lncRNAs’ most informative of recurrence from the differentially expressed lncRNAs. SVM–RFE is considered as an efficient method to select informative genes for cancer classification, in which all the features are listed based on some score function together with the removal of the features with the lowest scores [16]. One shortcoming of this method is that it only aims to identify the optimal combination for classification. RF is a highly data-adaptive classification tool based on decision trees that is especially suitable for high-dimension genomic data analysis with OBB error to assess the predictive performance of RF [28]. According to the results of PCA, the RF-OBB-based seven feature lncRNAs was more informative of recurrence in comparison with the SVM–RFE-based eight feature lncRNAs in the current study. From the seven feature lncRNAs, we identified a four-lncRNA signature that was significantly associated with patients’ RFS and had independent prognostic value. Moreover, a four-lncRNA risk score for outcome prediction was developed using TCGA set and validated using two GEO data sets. Our results indicated that this four-lncRNA risk score could successfully distinguish GC patients with high risk from GC patients with low risk. In addition, our study showed that predictive performance of the four lncRNAs-based risk score was independent of other clinical variables. Potential clinical application of this risk score would be beneficial to improving individualized treatment decision-making for GC patients.

The four identified prognostic lncRNAs were LINC00843, SNHG3, C21orf62-AS1, and MIR99AHG. lncRNA SNHG3 is reported to be implicated in development of various types of cancers, such as colorectal cancer and ovarian cancer [29,30]. SNHG3 is overexpressed in hepatocellular carcinoma (HCC), showing correlation with the survival of HCC patients [31]. SNHG3 is upregulated lncRNA in GC [32]. However, biological functions and prognostic value of SNHG3 in GC remain elusive. lncRNA C21orf62-AS1 is abnormally expressed in chromophobe renal cell carcinoma, correlating with OS of patients [33]. Additionally, C21orf62-AS is stimulated by interferon-beta in patients with multiple sclerosis [34]. lncRNA MIR99AHG is significantly downregulated in colorectal cancer [35]. There is evidence that MIR99AHG is positively relevant to OS of patients with lung squamous cell carcinoma [36]. There is little information concerning lncRNA LINC00843. As far as we know, this is the first time that these lncRNAs are found to be prognostic biomarkers for GC recurrence.

It has been demonstrated that ceRNAs act as key regulators among different RNA transcripts and lncRNAs sponges miRNAs, thereby regulating the expression of targeted mRNAs [37]. The present study established a ceRNA network comprising three signature lncRNAs (LINC00843, SNHG3, and MIR99AHG), 10 miRNAs, and 178 target mRNAs by bioinformatics prediction. Among the 10 miRNAs, miR-7 plays an antimetastatic role in GC through targeting insulin-like growth factor-1 receptor [38]. The miR-487a strengthens cell proliferation and suppresses cell apoptosis, driving GC progression by targeting T-cell intracellular antigen-1 [39]. Furthermore, three miRNAs including miR-216a, miR-205, and miR-551b are related to epithelial–mesenchymal transition (EMT) and metastasis of GC [40–42]. These findings are suggestive of implication of the three signature lncRNAs in metastasis of GC through influencing relevant miRNAs and target mRNAs. Noticeably, results of GO and KEGG pathway enrichment analysis demonstrated that mRNAs in the ceRNA network were associated with several Wnt-signaling pathway-related biological processes and KEGG-signaling pathway. Wnt-signaling pathway plays a fundamental role in progression and metastasis of GC, participating in regulating GC cell growth and apoptosis [43]. Involvement of Wnt-signaling pathway in EMT is controlled by miRNAs [44]. Besides, Wnt-signaling pathway is reported to mediate the oncogenic role of miR-552 by Dachshund family transcription factor 1 in colorectal cancer [45]. It can be speculated that Wnt-signaling pathway may partly mediate the effect of these signature lncRNAs on progression and metastasis of GC through miRNAs. Our study only contains results based on gene mining approaches. Clinical experimental studies and large prospective studies are necessary to verify our findings.

In conclusion, we generated a recurrence-related four-lncRNA signature predictive of individual mortality risk of DFS in GC patients. Prognostic capability of the lncRNAs-based signature had been successfully validated using two independent data sets and showed independence of other clinical features. The four-lncRNA signature functionally involved several metastasis-related miRNAs, their targeted mRNAs, and Wnt-signaling pathway. This study suggested potential prognostic biomarkers and therapeutic targets for recurrence GC and provided novel insights into the underlying mechanisms of GC progression. Validation of our findings and investigation on functional mechanisms warrant future studies.

## Abbreviations

GC: Gastric cancer
lncRNA: Long non-coding RNA
TCGA: The Cancer Genome Atlas
GEO: Gene Expression Omnibus
ceRNA: competing endogenous RNAs
miRNA: microRNA
OS: overall survival
HGNC: HUGO Gene Nomenclature Committee
DERs: differentially expressed RNAs
SVM: Support Vector Machine
RFE: Recursive Feature Elimination
RF: Random Forest
OOB: Out Of Bag
PCA: Principal Component Analysis
RFS: Recurrence-free survival
GO: Gene ontology
KEGG: kyoto encyclopedia of genes and genomes
CAMs: cell adhesion molecules
HCC: hepatocellular carcinoma
EMT: epithelial-mesenchymal transition
DACH: Dachshund family transcription factor
